# Impact of Different Operational Definitions on Mild Cognitive Impairment Rate and MMSE and MoCA Performance in Transient Ischaemic Attack and Stroke

**DOI:** 10.1159/000355496

**Published:** 2013-11-08

**Authors:** Sarah T. Pendlebury, Jose Mariz, Linda Bull, Ziyah Mehta, Peter M. Rothwell

**Affiliations:** ^a^Stroke Prevention Research Unit, Nuffield Department of Clinical Neurosciences, University of Oxford, Oxford, UK; ^b^NIHR Oxford Biomedical Research Centre, Oxford, UK

**Keywords:** Mild cognitive impairment, Montreal Cognitive Assessment, Mini Mental State Examination, Transient ischaemic attack, Stroke, Vascular cognitive impairment

## Abstract

**Background:**

Mild cognitive impairment (MCI) is at least as prevalent as dementia after transient ischaemic attack (TIA)/stroke and is increasingly recognised as an important outcome in observational studies and randomised trials. However, there is no consensus on how impairment should be defined, and numerous different criteria exist. Previous studies have shown that different criteria for cognitive impairment impact on prevalence rates in epidemiological studies. However, there are few data on how operational differences *within* established criteria (e.g. Petersen-MCI) affect measured impairment rates and the performance of short cognitive tests such as the Mini Mental State Examination (MMSE) and the Montreal Cognitive Assessment (MoCA), particularly in cerebrovascular disease. We therefore evaluated the effect of different operational definitions on measured rates of Petersen-MCI and on reliability of short cognitive tests in patients with TIA and stroke.

**Methods:**

Consecutive patients underwent the MMSE, MoCA and neuropsychological battery ≥1 year after TIA or stroke in a population-based study. MCI was defined using the Petersen method and subclassified as single or multiple domain, both with (original) and without (modified) subjective memory impairment. Different cut-offs (>1, >1.5 and >2 standard deviations, SD) on a given test relative to published norms were compared together with use of single versus multiple tests to define domain impairment.

**Results:**

91 non-demented subjects completed neuropsychological testing (mean age ± SD 69.7 ± 11.6 years, 54 male, 49 stroke) at a mean of 3.1 ± 1.9 years after the index event. Rates of cognitive impairment ranged from 14/91 (15%) for MCI-original at >2 SD cut-off to 61/91 (67%) MCI-modified at >1 SD cut-off, and the proportion of MCI that was multiple domain varied, e.g. 24/46 (52%) versus only 5/27 (20%) at 1 versus 2 SD cut-off for MCI-modified. Requirement for subjective memory complaint approximately halved estimates [e.g. 17 (19%) vs. 39 (43%) for MCI at 1.5 SD cut-off, single test definition], whereas use of multiple tests versus a single test to define a cognitive domain had less impact. In general, diagnostic accuracy was higher, and optimal cut-offs lower, on MMSE and MoCA for multiple-domain versus single-domain MCI, but the MoCA appeared superior for detecting MCI-modified, whereas the MMSE performed well in detecting MCI-original.

**Conclusion:**

Even within established criteria for MCI, differences in operational methodology result in 4-fold variation in MCI estimates. Optimal MMSE and MoCA cut-offs are lower, and reliability more similar, when criteria for MCI are more stringent. Our findings have implications for sample size and adjusted relative risk calculations in randomised trials and for comparisons between studies.

## Introduction

In the first year after stroke, around a quarter of patients will have dementia, rates being lowest in those with first ever stroke and highest in those with recurrent events [1,2]. Mild cognitive impairment (MCI) is at least as prevalent as dementia after transient ischaemic attack (TIA)/stroke [3] and is increasingly recognised as an important outcome in observational studies and randomised trials. However, there is no consensus on how impairment should be defined, and numerous different criteria exist. Previous studies have shown that the use of different criteria to define cognitive impairment without dementia affects reported prevalence rates in epidemiological studies [4,5,6,7], memory clinic subjects [8] and Parkinson's disease [9]. This is analogous to the findings of varying dementia estimates obtained across different dementia diagnostic criteria in patients with cerebrovascular disease [10,11].

However, even within established criteria for cognitive impairment such as those described by Petersen and colleagues [12], there is uncertainty around how such criteria should be applied in practice including the threshold at which abnormality should be set, i.e. 1 standard deviation (SD) or greater below normal performance [9], which norms are used as the standard, the definition of cognitive domains (i.e. which tests should be used and whether single or multiple tests are required) [9] and in defining subjective memory (or cognitive) impairment or even whether this should be a requirement [4].

Lack of consensus on how to operationalise established criteria is important since it is likely to result in lack of comparability between studies and conflicting results regarding the performance of short cognitive tests which is problematic since such short tests are increasingly used as outcome measures in large pragmatic studies and clinical trials. We hypothesised that lower thresholds for defining impairment would result in higher measured rates of cognitive impairment in patients with TIA and stroke, higher optimal cut-offs on short cognitive tests and superiority of the Montreal Cognitive Assessment (MoCA) [13], which is sensitive to milder cognitive deficits, over the Mini Mental State Examination (MMSE) which was designed to detect moderate/severe cognitive impairment [14]. Conversely, higher thresholds would result in lower measured rates and similar performance of MoCA and MMSE in detecting impairment. We therefore aimed to determine the effect of different operational definitions within the Petersen-MCI criteria [12], a widely used definition of cognitive impairment without dementia, on rates of MCI and reliability of short tests (MMSE and MoCA) ≥1 year after TIA and stroke.

## Methods

Patients were participants in the Oxford Vascular Study (OXVASC 2002 -), a prospective population-based cohort study of all acute vascular events occurring within a defined population of about 91,000 [15,16]. The study was approved by the local ethics committee (Oxfordshire Clinical Research Ethics Committee, CO.043), and informed consent was obtained. Between August 2009 and November 2010, consecutive patients were invited at their routine 1- or 5-year follow-up to undergo further cognitive testing with the National Institute of Neurological Disorders and Stroke-Canadian Stroke Network (NINDS-CSN) Vascular Cognitive Impairment Harmonization Standards Neuropsychological Battery [17] in addition to the MMSE [14] and MoCA [13] which were done routinely at the follow-up appointment. Some data on this cohort have been reported previously [3,18]. Further cognitive testing was performed by investigators blinded to the MMSE and MoCA results. Patients who had problems that interfered with testing, such as poor vision, severe hearing impairment, inability to use the right arm, dysphasia, poor English or acute illness, were excluded as described previously [19].

The NINDS-CSN Harmonization Standards [17] recognised that there are no perfect neuropsychological tests but recommended tests based on their wide usage, availability of published norms and ability to detect impairment in ‘executive/activation function, processing speed, word retrieval and episodic memory’ of particular importance in vascular cognitive impairment. The battery took around 50-60 min to administer and included:

(i) Trail Test (parts A and B) [20,21];

(ii) Symbol Digit Modalities Test [22];

(iii) Boston Naming Test (30-item version) [21,23];

(iv) Rey-Osterrieth complex figure copy [24,25];

(v) Hopkins Verbal Learning Test-Revised [26,27];

(vi) letter (Controlled Oral Word Association Test [21,28] and category (animals) fluency [29].

The presence of a subjective memory complaint was examined using the question: ‘Do you think you have more problems with your memory than most?’ Although ‘subjective memory complaint’ was broadened to ‘subjective cognitive complaint’ in the 2004 publication on the MCI criteria [12], we chose to identify memory rather than ‘cognitive’ complaints owing to uncertainties in defining the latter in practice.

For MCI by the Petersen method, 4 subtypes of MCI were distinguished [12]:

(i) amnestic single domain: objective impairment of memory only;

(ii) amnestic multiple domain: memory and at least one other cognitive domain impaired;

(iii) non-amnestic single domain: one single domain other than memory impaired;

(iv) non-amnestic multiple domain: at least two cognitive domains impaired but not memory.

We examined the effect of different operational methodologies as follows:

(i) different thresholds (1, 1.5 and 2 SD below published norms as used in previous studies [9]) for defining impairment on a given test corrected for age and education;

(ii) using abnormal performance on a single test versus combinations of tests to define impairment in a given cognitive domain [4,9] (online suppl. methods and table S1; for all online suppl. material, see www.karger.com/doi/10.1159/000355496); test combinations for a given cognitive domain were selected on the basis of the NINDS-CSN Harmonization Standards recommendations [17];

(iii) requiring multiple-versus single-domain impairment;

(iv) with (Petersen-original criteria) and without (Petersen-modified criteria) requirement for a subjective memory complaint [4].

For all designations of cognitive impairment, subjects had no impairment of basic functional activities of daily living as measured by the Barthel index, and did not fulfil the DSM-IV dementia diagnostic criteria [30].

### Statistical Analyses

An additional point for low education (≤12 years) was added to the MoCA score in line with the original publication [13]. Reliability of MMSE and MoCA for MCI diagnosis by the different operational criteria was assessed using the area under the receiver-operating characteristic curve (AUC). AUC were calculated for all patients with any MCI (both single- and multiple-domain MCI) versus no MCI and then separately for those with single-domain MCI versus no MCI, multiple-domain MCI versus no MCI + single-domain MCI, multiple-domain MCI versus single-domain MCI and multiple-domain MCI versus no MCI for each operational definition. Sensitivities and specificities for various MoCA and MMSE cut-offs for identifying cognitive impairment were determined from the AUC curves.

## Results

Ninety-one non-demented patients completed MMSE, MoCA and Neuropsychological Battery assessments at a mean of 3.1 ± 1.9 years after the index event. TIA (n = 42) and stroke (n = 49) patients were similar in age (69.8 ± 9.0 vs. 69.7 ± 13.6 years, p = 0.97), male sex (55 vs. 63%, p = 0.52) and education <12 years (67 vs. 59%, p = 0.52).

**Fig. 1:**
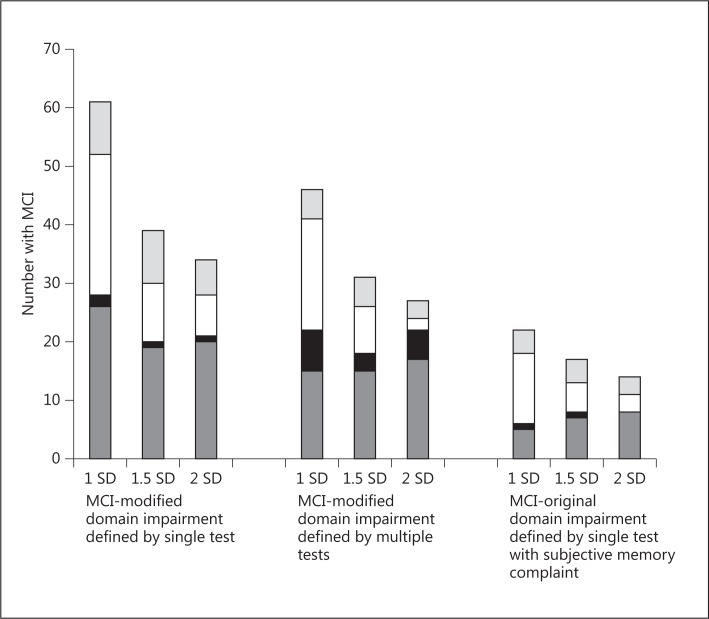
Total numbers with MCI and with the different MCI subtypes (dark grey = non-amnestic single-domain, black = amnestic single-domain, white = amnestic multiple-domain, light grey = non-amnestic multiple-domain) for the different operational definitions of MCI.

Rates of cognitive impairment ranged from 14/91 (15%) for the most stringently defined cognitive impairment (MCI-original at 2 SD cut-off) to 61/91 (67%) for the least stringently defined impairment (MCI-modified, single test definition, at the 1 SD cut-off; table 1). MCI was more likely to be classed as multiple domain when criteria for impairment were less stringent, e.g. within the MCI-modified, multiple test definition, multiple-domain MCI was 24/46 (52%) at 1 SD cut-off compared to only 5/27 (20%) at 2 SD cut-off (table 1). Amnestic single-domain impairment was relatively rare irrespective of methodology ranging from 0 to 20% of total MCI (fig. 1; table 1). Regardless of the chosen cut points for defining abnormality (i.e. number of SD below norm), requirement for a subjective memory complaint at least halved the measured rate of impairment using the single test definition: 17 (19%) MCI-original versus 39 (43%) MCI-modified, at 1.5 SD cut-off. In contrast, use of a single test versus multiple tests to define cognitive domain impairment had less impact, although rates were lower when multiple tests were used (table 1, fig. 1).

Looking at TIA and stroke patients separately, MCI was more likely in stroke versus TIA patients: 27/49 (55%) versus 12/42 (29%), with OR = 3.06, 95% CI = 1.28-7.36, p = 0.01 for MCI-modified, 1.5 SD cut-off and single test definition, and 12/49 (24%) versus 5/42 (12%), with OR = 2.40, 95% CI = 0.77-7.50, p = 0.13 for MCI-original, 1.5 SD cut-off (online suppl. table S2). Multiple-domain MCI was more likely than single-domain MCI in stroke patients, whereas the opposite was seen in TIA patients.

AUC were generally higher for the MoCA than the MMSE for detecting MCI where subjective memory complaint was not required (MCI-modified), whereas the MMSE was more reliable at identifying MCI-original. More stringent cut-offs (1.5 or 2 SD) for defining MCI usually resulted in higher AUC. AUC were also higher for identifying multiple-domain MCI than for identifying single-domain MCI and were highest for multiple-domain MCI versus no MCI, i.e. when the difference in cognitive performance between the two groups being compared was greatest (table 2). Both MMSE and MoCA performed least well in discriminating between single-domain MCI and no MCI and between single- and multiple-domain MCI, i.e. when the difference between the two groups being compared was small, although AUC for MoCA were higher than for MMSE for MCI-modified. Although numbers were too small to be precise about the relative performance of the MMSE and MoCA for detecting MCI separately in TIA and stroke patients, AUC values were broadly similar (online suppl. table S3).

Optimal cut-offs ranged from MMSE <30 to <26 and MoCA <27 to <23 and were lower when requirements for cognitive impairment were more stringent, i.e. for MCI defined using 1.5 or 2 rather than 1 SD cut-offs below the norm or as multiple-domain impairment (table 3). The MMSE had low sensitivity for mild impairment compared to the MoCA with evidence of a marked ceiling effect.

## Discussion

Operational differences in applying Petersen-MCI criteria in patients after TIA and stroke have a major impact on measured rates of impairment and on the relative proportion of single-versus multiple-domain MCI and also on performance of short cognitive tests. Varying the threshold used to define abnormality and requiring a subjective memory complaint had greater impact than the use of a single versus multiple tests to define abnormality in a given domain.

Previous studies have described variable estimates of (vascular) dementia and cognitive impairment *across* different diagnostic criteria [4,5,6,7,8,9,10,11], but our study provides the first data on the impact of operational methodology on the measured rate of cognitive impairment without dementia *within* a given set of criteria (Petersen-MCI) in patients with cerebrovascular disease and on optimal cut-offs on short cognitive tests. We observed that the measured rate of MCI after TIA and stroke was highly dependent on operational methodology with lowest rates seen when cut-offs were most stringent, in keeping with studies in Parkinson's disease [9].

Amnestic single-domain impairment, common in memory research cohorts, was rare, in keeping with findings from clinical practice [8], whereas non-amnestic single-domain impairment was prevalent regardless of operational methodology. However, the relative proportions of MCI subtypes and in particular of multiple-versus single-domain impairment depended on the operational criteria used to define MCI with more stringent cut-offs reducing the proportion of multi-domain impairment. Although multiple-versus single-domain impairment has been shown to be associated with a greater risk of conversion to dementia on follow-up [31,32], our results suggest that this risk will vary according to the stringency of the MCI definition.

Regardless of operational definition, multiple-domain cognitive impairment was generally detected more reliably than single-domain impairment by short cognitive tests particularly by the MMSE perhaps unsurprisingly, since the MMSE was developed to detect moderate/severe cognitive impairment. The MMSE also performed well in detecting MCI-original (subjective memory complaint required) suggesting sensitivity to Alzheimer-type impairment presumably owing to an emphasis on memory, language and orientation. When subjective memory complaint was not a requirement, MCI rates were much higher and the MoCA performed well in detecting both single- and multiple-domain impairment consistent with its sensitivity to mild deficits not detected by the MMSE [9,20]. These findings suggest that use of MCI-modified and the MoCA rather than the MMSE are appropriate for studies of vascular cognitive impairment.

Given the reliability of short cognitive tests for detection of multi-domain impairment, short tests may be equally as good as the formal Neuropsychological Battery in identifying those at high risk of conversion to dementia. Further, it has been shown that some subjects with global cognitive impairment defined on short tests do not have MCI by formal criteria but are nevertheless at high risk of dementia [33]. If short cognitive tests are shown to have good prognostic value, this will be of great value in routine clinical practice and large studies where long neuropsychological batteries are not feasible.

Our results provide some explanation for conflicting results from previous studies regarding optimal cut-offs and relative performance of the MMSE and MoCA for detection of cognitive impairment after stroke. Godefroy et al. [34] performed the MoCA in the acute phase of stroke and compared it with the Neuropsychological Battery around 3 weeks later and found optimal cut-offs in the low 20s and that the specificity of the MoCA was moderate, the latter performing no better overall than the MMSE. Dong et al. [35] found optimal MoCA cut-offs of 21/22 when administered acutely for predicting cognitive impairment at 3-6 months. However, we found, in stable patients with TIA and stroke, that optimal MoCA cut-offs were around 25 and that the MoCA was more sensitive than the MMSE for milder deficits whilst retaining reasonable specificity [3].

The relatively low MoCA cut-offs and similar MMSE and MoCA performance in the studies by Godefroy et al. [34] and Dong et al. [35] could be explained in part by the requirement for multiple-domain/moderate to severe impairment, whereas we included single-domain impairment – our optimal MoCA and MMSE cut-offs for detecting multiple-domain impairment are not that dissimilar. Also, the Godefroy and Dong studies used the MoCA acutely with neuropsychological evaluation some weeks/months later, and significant cognitive recovery is likely to have occurred between testing points – even minor cerebrovascular events are known to cause transient cognitive impairment even in the absence of overt delirium [36]. Lastly, we included TIA patients as well as those with stroke, whereas the Dong and Godefroy studies were confined to stroke patients.

Our study has some limitations. First, we used a relatively small sample of stable patients with TIA and stroke, and patients with dysphasia and severe stroke were not included. Second, we did not determine the effect of informant report of cognitive decline on the measured rate of impairment. Third, we were not able to examine the effects of different psychological tests and norms within a given domain owing to the need to be pragmatic in testing older and sometimes frail patients, but this is likely to be an additional factor in comparing results between studies. Finally, we selected tests according to the Harmonisation Standards recommendations [17] which are biased towards verbal functioning and thus non-verbal functions, particularly memory, were not assessed in detail in our study and this may have impacted on the findings.

In conclusion, even within established criteria for MCI, differences in operational definitions resulted in 4-fold variation in MCI estimates. In addition, optimal MMSE and MoCA cut-offs were lower, and reliability of the two tests was more similar, when requirements for MCI were more stringent. Our findings have implications for measuring cognitive impairment outcomes in randomised trials and observational studies in terms of sample size and adjusted relative risk calculations and choice of cut-offs on short cognitive tests. Our data should also facilitate the interpretation of reported MCI rates from across different studies.

## Disclosure Statement

Competing interests: none declared.

## Supplementary Material

Supplementary data

## Figures and Tables

**Table 1:** Effect of different operational definitions on numbers with MCI in the total sample (n = 91) and numbers with single-versus multiple-domain and amnestic versus non-amnestic impairment and thus of the four MCI subtypes (amnestic single-domain, non-amnestic single-domain, amnestic multiple-domain and non-amnestic multiple-domain)

	MCI-modified, using single test			MCI-modified, using multiple tests			MCI-original, using single test		
	1 SD	1.5 SD	2 SD	1 SD	1.5 SD	2 SD	1 SD	1.5 SD	2 SD
Total MCI, n of sample	61 (67%)	39 (43%)	34 (37%)	46 (51%)	31 (34%)	27 (30%)	22 (24%)	17 (19%)	14 (15%)
Single-domain MCI	28	20	21	22	18	22	6	8	8
Amnestic	2	1	1	7	3	5	1	1	0
Non-amnestic	26	19	20	15	15	17	5	7	8
Multiple-domain MCI	33	19	13	24	13	5	16	9	6
Amnestic	24	10	7	19	8	2	12	5	3
Non-amnestic	9	9	6	5	5	3	4	4	3

**Table 2:** Effect of different MCI operational definitions on AUC for MMSE and MoCA for any MCI (single- and multiple-domain MCI) versus no MCI, single-domain MCI versus no MCI, multiple-domain MCI versus no MCI + single-domain MCI and multiple-domain MCI versus no MCI

	MCI-modified, using single test			MCI-modified, using multiple tests			MCI-original, using single test		
	1 SD	1.5 SD	2 SD	1 SD	1.5 SD	2 SD	1 SD	1.5 SD	2 SD
AUC any (single- and multiple-domain) MCI versus no MCI									
MMSE	0.76	0.83	0.85	0.78	0.83	0.84	0.74	0.82	0.88
MoCA	0.79	0.85	0.84	0.80	0.85	0.83	0.71	0.79	0.80

AUC single-domain MCI versus no MCI									
MMSE	0.65	0.74	0.83	0.69	0.76	0.82	0.69	0.71	0.88
MoCA	0.70	0.78	0.80	0.70	0.78	0.80	0.64	0.72	0.77

AUC multiple-domain MCI versus no MCI + single-domain MCI									
MMSE	0.79	0.89	0.80	0.84	0.88	0.84	0.75	0.91	0.86
MoCA	0.82	0.90	0.84	0.88	0.88	0.89	0.73	0.86	0.82

AUC multiple-domain MCI versus single-domain MCI									
MMSE	0.71	0.81	0.60	0.77	0.72	0.61	0.58	0.83	0.65
MoCA	0.75	0.82	0.67	0.83	0.75	0.72	0.63	0.82	0.66

AUC multiple-domain MCI versus no MCI									
MMSE	0.86	0.93	0.88	0.87	0.92	0.92	0.76	0.92	0.88
MoCA	0.87	0.93	0.90	0.90	0.93	0.95	0.73	0.86	0.84

**Table 3:** Effect of different MCI operational definitions on MMSE and MoCA optimal cut-offs, sensitivity and specificity

	MCI-modified, using single test			MCI-modified, using multiple tests			MCI-original, using single test		
	1 SD	1.5 SD	2 SD	1 SD	1.5 SD	2 SD	1 SD	1.5 SD	2 SD
Optimal cut-off, sensitivity, specificity: any (single− + multiple-domain) MCI versus no MCI									
MMSE	<30, 84, 43	<29, 77, 81	<29, 79, 77	<30, 91, 42	<29, 81, 65	<29, 82, 72	<29, 77, 67	<29, 88, 66	<29, 93, 65
	<29, 59, 87	<28, 64, 88	<28, 68, 86	<29, 67, 80	<28, 65, 82	<28, 67, 80	<28, 68, 67	<28, 82, 77	<28, 93, 67
MoCA	<27, 85, 60	<26, 87, 64	<26, 85, 58	<27, 91, 51	<26, 84, 55	<26, 82, 52	<26, 85, 51	<25, 82, 66	<25, 86, 65
	<26, 71, 67	<25, 77, 83	<25, 79, 79	<26, 78, 38	<25, 81, 67	<25, 78, 72	<25, 68, 65	<24, 71, 74	<24, 71, 73

Optimal cut-off, sensitivity, specificity: single-domain MCI versus no MCI									
MMSE	<30, 71, 43	<30, 90, 40	<29, 76, 77	<30, 88, 42	<30, 94, 37	<29, 77, 72	<30, 83, 29	<29, 75, 66	<29, 100, 65
	<29, 39, 87	<29, 60, 81	<28, 67, 86	<29, 45, 80	<29, 67, 75	<28, 64, 80	<29, 67, 67	<28, 75, 77	<28, 100, 77
MoCA	<28, 82, 43	<27, 90, 48	<27, 90, 44	<28, 91, 38	<28, 94, 32	<27, 91, 39	<27, 83, 36	<26, 100, 51	<26, 100, 49
	<27, 75, 60	<26, 80, 63	<26, 81, 58	<27, 86, 51	<27, 89, 42	<26, 77, 52	<26, 83, 51	<25, 75, 66	<25, 88, 65

Optimal cut-off, sensitivity, specificity: multiple-domain MCI versus no MCI + single-domain MCI									
MMSE	<29, 76, 74	<29, 95, 69	<29, 85, 63	<29, 88, 62	<28, 85, 74	<28, 80, 69	<29, 88, 48	<28, 89, 72	<27, 83, 78
	<28, 61, 81	<28, 79, 78	<28, 69, 72	<28, 71, 79	<27, 69, 81	<27, 80, 77	<28, 69, 73	<27, 78, 79	<26, 83, 85
MoCA	<26, 85, 57	<25, 90, 69	<25, 85, 64	<25, 88, 73	<25, 92, 65	<24, 100, 60	<26, 88, 48	<25, 89, 62	<25, 83, 60
	<25, 69, 63	<24, 92, 76	<24, 77, 73	<24, 61, 81	<24, 92, 76	<23, 80, 77	<25, 67, 63	<24, 89, 72	<24, 83, 69

Optimal cut-off, sensitivity, specificity: multiple-domain MCI versus single-domain MCI									
MMSE	<29, 76, 61	<29, 95, 40	<27, 62, 57	<29, 88, 55	<28, 85, 50	<27, 80, 50	<28, 69, 33	<27, 78, 50	<27, 83, 38
	<28, 61, 68	<28, 79, 50	<26, 62, 71	<28, 71, 64	<27, 69, 56	<26, 80, 59	<27, 50, 33	<26, 78, 88	<26, 83, 50
MoCA	<26, 85, 46	<24, 84, 65	<24, 77, 48	<25, 88, 59	<24, 92, 50	<24, 100, 41	<25, 69, 33	<24, 89, 50	<24, 83, 38
	<25, 76, 64	<23, 74, 75	<23, 69, 57	<24, 79, 77	<23, 77, 56	<23, 80, 45	<24, 63, 50	<23, 67, 75	<23, 67, 73

Optimal cut-off, sensitivity, specificity: multiple-domain MCI versus no MCI									
MMSE	<30, 94, 43	<29, 95, 81	<29, 85, 77	<30, 96, 42	<29, 100, 75	<28, 80, 80	<29, 81, 67	<28, 89, 77	<28, 83, 77
	<29, 76, 87	<28, 79, 88	<28, 69, 86	<29, 88, 80	<28, 85, 82	<27, 80, 86	<28, 69, 77	<27, 78, 82	<27, 83, 82
MoCA	<27, 94, 60	<26, 95, 63	<26, 92, 58	<27, 96, 51	<26, 92, 55	<24, 100, 80	<26, 85, 52	<26, 100, 51	<26, 100, 49
	<26, 85, 67	<25, 89, 83	<25, 85, 79	<26, 92, 62	<25, 92, 77	<23, 80, 88	<25, 69, 65	<25, 89, 66	<25, 83, 65

Two alternative adjacent cut-offs that appear to offer the best balance between sensitivity and specificity are given.

## References

[1] Pendlebury ST, Rothwell PM (2009). Prevalence, incidence, and factors associated with pre-stroke and post-stroke dementia: a systematic review and meta-analysis. Lancet Neurol.

[2] Pendlebury ST, Rothwell PM (2009). Risk of recurrent stroke, other vascular events and dementia after transient ischaemic attack and stroke. Cerebrovasc Dis.

[3] Pendlebury ST, Mariz J, Bull L, Mehta Z, Rothwell PM (2012). MoCA, ACE-R, and MMSE versus the National Institute of Neurological Disorders and Stroke-Canadian Stroke Network Vascular Cognitive Impairment Harmonization Standards Neuropsychological Battery after TIA and stroke. Stroke.

[4] Busse A, Bischkopf J, Riedel-Heller SG, Angermeyer MC (2003). Subclassifications for mild cognitive impairment: prevalence and predictive validity. Psychol Med.

[5] Fisk JD, Merry HR, Rockwood K (2003). Variations in case definition affect prevalence but not outcomes of mild cognitive impairment. Neurology.

[6] Stephan BCM, Matthews FE, McKeith IG, Bond J, Brayne C, Medical Research Council Cognitive Function and Aging Study (2007). Early cognitive change in the general population: how do different definitions work?. J Am Geriatr Soc.

[7] Ward A, Arrighi HM, Michels S, Cedarbaum JM (2012). Mild cognitive impairment: disparity of incidence and prevalence estimates. Alzheimers Dement.

[8] Alladi S, Arnold R, Mitchell J, Nestor PJ, Hodges JR (2006). Mild cognitive impairment: applicability of research criteria in a memory clinic and characterization of cognitive profile. Psychol Med.

[9] Liepelt-Scarfone I, Graeber S, Feseker A, Baysal G, Godau J, Gaenslen A, Maetzler W, Berg D (2011). Influence of different cut-off values on the diagnosis of mild cognitive impairment in Parkinson's disease. Parkinsons Dis.

[10] Pohjasvaara T, Erkinjuntti T, Vataja R, Kaste M (1997). Dementia three months after stroke: baseline frequency and effect of different definitions of dementia in the Helsinki Stroke Aging Memory Study (SAM) cohort. Stroke.

[11] Pohjasvaara T, Mäntylä R, Ylikoski R, Kaste M, Erkinjuntti T (2000). Comparison of different clinical criteria (DSM-III, ADDTC, ICD-10, NINDS-AIREN, DSM-IV) for the diagnosis of vascular dementia. National Institute of Neurological Disorders and Stroke-Association Internationale pour la Recherche et l'Enseignement en Neurosciences. Stroke.

[12] Winblad B, Palmer K, Kivipelto M, Jelic V, Fratiglioni L, Wahlund LO (2004). Mild cognitive impairment: beyond controversies, towards a consensus – report of the International Working Group on Mild Cognitive Impairment. J Intern Med.

[13] Nasreddine ZS, Phillips NA, Bedirian V, Charbonneau S, Whitehead V, Collin I (2005). The Montreal Cognitive Assessment, MoCA: a brief screening tool for mild cognitive impairment. J Am Geriatr Soc.

[14] Folstein MF, Folstein SE, McHugh PR (1975). ‘Mini-mental state’. A practical method for grading the cognitive state of patients for the clinician. J Psychiatr Res.

[15] Rothwell PM, Coull AJ, Giles MF, Howard SC, Silver LE, Bull LM (2004). Oxford Vascular Study: change in stroke incidence, mortality, case-fatality, severity, and risk factors in Oxfordshire, UK, from 1981 to 2004 (Oxford Vascular Study). Lancet.

[16] Rothwell PM, Coull AJ, Silver LE, Fairhead JF, Giles MF, Lovelock CE (2005). Oxford Vascular Study: population-based study of event-rate, incidence, case fatality, and mortality for all acute vascular events in all arterial territories (Oxford Vascular Study). Lancet.

[17] Hachinski V, Iadecola C, Petersen RC, Breteler MM, Nyenhuis DL, Black SE (2006). National Institute of Neurological Disorders and Stroke-Canadian Stroke Network vascular cognitive impairment harmonization standards. Stroke.

[18] Pendlebury ST, Welch SJ, Cuthbertson FC, Mariz J, Mehta Z, Rothwell PM (2013). Telephone assessment of cognition after transient ischemic attack and stroke: modified telephone interview of cognitive status and telephone Montreal Cognitive Assessment versus face-to-face Montreal Cognitive Assessment and neuropsychological battery. Stroke.

[19] Pendlebury ST, Cuthbertson FC, Welch SJ, Mehta Z, Rothwell PM (2010). Underestimation of cognitive impairment by Mini-Mental State Examination versus the Montreal Cognitive Assessment in patients with transient ischemic attack and stroke. A population-based study. Stroke.

[20] Reitan R (1955). The relation of the trail making test to organic brain damage. J Consult Psychol.

[21] Ivnik R, Malec J, Smith G, Tangalos E, Petersen R (1996). Neuropsychological tests' norms above age 55: COWAT, BNT, MAE TOKEN, WRAT-R READING, AMNART, Stroop, TMT and JLO. Clin Neuropsychol.

[22] Smith A (1982). Symbol Digit Modalities Test (SDMT): Manual, rev.

[23] Franzen MD, Haut MW, Rankin E, Keefover R (1995). Empirical comparison of alternate forms of the Boston Naming Test. Clin Neuropsychol.

[24] Corwin J, Bylsma FW (1993). Psychological examination of traumatic encephalopathy. Clin Neuropsychol.

[25] Fasteneau PS, Denburg NL, Hufford BJ (1999). Adult norms for the Rey-Osterrieth Complex Figure Test and for supplemental recognition and matching trials from the Extended Complex Figure Test. Clin Neuropsychol.

[26] Brandt J (1991). The Hopkins Verbal Learning Test: development of a new memory test with six equivalent forms. Clin Neuropsychol.

[27] Benedict RHB, Schretlen D, Groninger L, Brandt J (1998). Hopkins Verbal Learning Test – Revised: normative data and analysis of inter-form and test-retest reliability. Clin Neuropsychol.

[28] Benton AL, Hamsher K (1994). Multilingual Aphasia Examination.

[29] Isaacs B, Kennie AT (1973). The set test as an aid to the detection of dementia in old people. Br J Psychiatry.

[30] American Psychiatric Association (1994). Diagnostic and Statistical Manual of Mental Disorders (DSM-IV).

[31] Ritchie LJ, Tuokko H (2010). Patterns of cognitive decline, conversion rates, and predictive validity for 3 models of MCI. Am J Alzheimers Dis Other Dement.

[32] Narasimhalu K, Ang S, De Silva DA, Wong MC, Chang HM, Chia KS (2009). Severity of CIND and MCI predict incidence of dementia in an ischemic stroke cohort. Neurology.

[33] Palmer K, Bäckman L, Winblad B, Fratiglioni L (2008). Mild cognitive impairment in the general population: occurrence and progression to Alzheimer disease. Am J Geriatr Psychiatry.

[34] Godefroy O, Fickl A, Roussel M, Auribault C, Bugnicourt JM, Lamy C (2011). Is the Montreal Cognitive Assessment superior to the Mini-Mental State Examination to detect poststroke cognitive impairment?. A study with neuropsychological evaluation. Stroke.

[35] Dong Y, Sharma VK, Chan BP, Venketasubramanian N, Teoh HL, Seet RC, Tanicala S, Chan YH, Chen C (2010). The Montreal Cognitive Assessment (MoCA) is superior to the Mini-Mental State Examination (MMSE) for the detection of vascular cognitive impairment after acute stroke. J Neurol Sci.

[36] Pendlebury ST, Wadling S, Silver LE, Mehta Z, Rothwell PM (2011). Transient cognitive impairment in TIA and minor stroke. Stroke.

