# Supplementary material for: Impact of Different Operational Definitions on Mild Cognitive Impairment Rate and MMSE and MoCA Performance in Transient Ischaemic Attack and Stroke
Source: Cerebrovasc Dis. 2013 Nov 8;36(5-6):355–62. doi: 10.1159/000355496 (PMC3902763; doi:10.1159/000355496)
Supplement: Supplementary file 1 — Supplementary data [file ced-0036-0355-s01.doc]

**Impact of different operational definitions on MCI rate and MMSE and MoCA performance in TIA and stroke**

Sarah T Pendlebury MRCP DPhil,^1,2^ Jose Mariz MD,^1^ Linda Bull RGN,^1^ Ziyah Mehta DPhil,^1^ Peter M Rothwell FRCP FMedSci^1^

^1^Stroke Prevention Research Unit, University Department of Clinical Neurology, John Radcliffe Hospital, and the University of Oxford

^2^NIHR Biomedical Research Centre, John Radcliffe Hospital, Oxford

**Supplementary Methods**

Tests for the neuropsychological battery were selected according to those recommended in the 60 minute protocol in the National Institute of Neurological Disorders and Stroke-Canadian Stroke Network Vascular Cognitive Impairment Harmonization Standards, Stroke 2006;37:2220-2241. This paper recommends tests in four domains “Executive/activation, language, visuospatial and memory” and recognises that there are no perfect tests. The Harmonisation Standards suggest that the recommended tests may be combined to further assess cognitive domains (Supplementary table 1).

For the MCI-single test definition, patients were divided into those with and without memory impairment on the HVLT immediate or delayed recall. Patients with impairment on the HVLT were then classified as MCI amnestic single-domain (no other tests abnormal) or amnestic multiple-domain (>1 other test abnormal) (Supplementary Table S1). Patients without impairment on the HVL were classified as non-amnestic single domain if they were impaired on a single non-memory test or non-amnestic multiple-domain if more than one non-memory test was impaired.

For the MCI-multiple tests definition, patients were divided into those with and without memory impairment on the HVLT immediate and delayed recall. Patients with impairment on the HVLT were then classified as amnestic single-domain (no other domain abnormal) or amnestic multiple domain if a non-memory domain was also impaired (see Supplementary table). Patients without impairment on the HVL were classified as non-amnestic single domain if they were impaired in one non-memory domain (see Supplementary table) or non-amnestic multiple-domain if more than one non-memory domain was impaired.

Supplementary Table S1. Operational methodology for defining MCI including sub-types for cognitive domains defined using a single test and multiple tests.

|  | MCI –single test definition | MCI-multiple test definition |
| --- | --- | --- |
| Amnestic-single | HVLT immediate or delayed recall impaired. No other abnormal test. | Memory impaired (HVLT immediate and delayed recall impaired). No other abnormal domain. |
| Non-amnestic single | Any one non-memory test impaired, no other abnormal test | Memory not impaired (HVLT normal)  One of the following abnormal:  Executive/activation function (> /= 2 abnormal tests of verbal fluency, symbol digit modalities test, Trail making test)  OR  Language (Boston naming test or verbal fluency)  OR  Visuo-spatial function (Rey-Osterrieth figure copy) |
| Amnestic-multiple | HVLT immediate or delayed recall impaired and >1 other test | Memory abnormal (HVLT) and at least one other of:  Executive/activation function (> /= 2 abnormal tests of verbal fluency, symbol digit modalities test, Trail making test)  OR  Language (Boston naming test or verbal fluency)  OR  Visuo-spatial function (Rey-Osterrieth figure copy) |
| Non-amnestic multiple | HVLT not impaired but >1 other test impaired | Memory not impaired (HVLT normal) and more than one of the following abnormal:  Executive/activation function (> /= 2 abnormal tests of verbal fluency, symbol digit modalities test, Trail making test)  OR  Language (Boston naming test or verbal fluency)  OR  Visuo-spatial function (Rey-Osterrieth figure copy) |

Supplementary Table.S1. Effect of different operational definitions on numbers with MCI in patients with TIA (n=42) and stroke (n=49) and numbers with single versus multiple domain impairment and of the four MCI-subtypes (amnestic single-domain, non-amnestic single domain, amnestic multiple domain and non-amnestic multiple domain). Results shown for 1.5SD cut-off and single test definition for MCI.

Table 2. Rates of MCI in TIA and stroke patients.

|  | **TIA n=42** | | **Stroke n=49** | |
| --- | --- | --- | --- | --- |
|  | **MCI-modified, using single test** | **MCI-original, using single test** | **MCI-modified, using single test** | **MCI-original, using single test** |
| **Total MCI**  N (%) of sample | 12 (29) | 5 (12) | 27 (55) | 12 (24) |
|  |  |  |  |  |
| **Single domain MCI** | 7 | 3 | 13 | 5 |
| Amnestic | 0 | 0 | 1 | 1 |
| Non-amnestic | 7 | 3 | 12 | 4 |
|  |  |  |  |  |
| **Multiple domain MCI** | 5 | 2 | 14 | 7 |
| Amnestic | 2 | 1 | 8 | 4 |
| Non-amnestic | 3 | 1 | 6 | 3 |

Supplementary Table S2. Effect of different MCI operational definitions at 1.5 SD cut-off, single test definition on AUC for MMSE and MoCA for TIA and stroke patients.

|  | TIA | | Stroke | |
| --- | --- | --- | --- | --- |
|  | MCI-modified | MCI-original | MCI-modified | MCI-original |
| AUC Single+ multiple domain MCI | | | | |
| MMSE | 0.839 | 0.814 | 0.829 | 0.820 |
| MoCA | 0.829 | 0.822 | 0.857 | 0.752 |
| AUC Multiple domain only | | | | |
| MMSE | 0.914 | 0.881 | 0.873 | 0.915 |
| MoCA | 0.846 | 0.813 | 0.901 | 0.850 |
